# Incorporating Field Studies into Species Distribution and Climate Change Modelling: A Case Study of the Koomal *Trichosurus vulpecula hypoleucus* (Phalangeridae)

**DOI:** 10.1371/journal.pone.0154161

**Published:** 2016-04-22

**Authors:** Shaun W. Molloy, Robert A. Davis, Eddie J. B. van Etten

**Affiliations:** School of Natural Sciences, Edith Cowan University, Joondalup, Western Australia, Australia; University of Saskatchewan, CANADA

## Abstract

Species distribution models (SDMs) are an effective way of predicting the potential distribution of species and their response to environmental change. Most SDMs apply presence data to a relatively generic set of predictive variables such as climate. However, this weakens the modelling process by overlooking the responses to more cryptic predictive variables. In this paper we demonstrate a means by which data gathered from an intensive animal trapping study can be used to enhance SDMs by combining field data with bioclimatic modelling techniques to determine the future potential distribution for the koomal (*Trichosurus vulpecula hypoleucus*). The koomal is a geographically isolated subspecies of the common brushtail possum, endemic to south-western Australia. Since European settlement this taxon has undergone a significant reduction in distribution due to its vulnerability to habitat fragmentation, introduced predators and tree/shrub dieback caused by a virulent group of plant pathogens of the genus *Phytophthora*. An intensive field study found: 1) the home range for the koomal rarely exceeded 1 km in in length at its widest point; 2) areas heavily infested with dieback were not occupied; 3) gap crossing between patches (>400 m) was common behaviour; 4) koomal presence was linked to the extent of suitable vegetation; and 5) where the needs of koomal were met, populations in fragments were demographically similar to those found in contiguous landscapes. We used this information to resolve a more accurate SDM for the koomal than that created from bioclimatic data alone. Specifically, we refined spatial coverages of remnant vegetation and dieback, to develop a set of variables that we combined with selected bioclimatic variables to construct models. We conclude that the utility value of an SDM can be enhanced and given greater resolution by identifying variables that reflect observed, species-specific responses to landscape parameters and incorporating these responses into the model.

## Introduction

Species Distribution Models (SDMs) encompass a broad and growing suite of tools and methods that enable the user to spatially define and analyse the potential distribution of target species and ecological assemblages [1, 2]. They have the capacity to demonstrate the potential consequences of human impacts and/or management actions by quantifying the spatial relationships between populations and the variables that define or reflect their habitat preferences [3]. Consequently, SDMs can be particularly useful in informing conservation management [4, 5].

To date, SDMs using bioclimatic envelopes have been the principal approach used to project changes in the potential distribution of species as a consequence of climatic change [6]. The reliability of SDMs has been questioned [1, 6, 7], but studies have shown that when properly undertaken, they are capable of determining the bioclimatically-defined potential distributions of biota and how these potential distributions will change in response to predicted climate change based on accepted global climate models (GCMs) [8, 9]. In this capacity, SDMs have been able to effectively quantify climate change impacts on many species and communities [10–15]. The use of bioclimatic variables as sole predictors has proven effective at very broad scales (i.e. large regions to continental),however this form of data is usually produced at a coarse level of resolution and consequently often lacks the ability to determine habitat requirements and distributions at finer scales [16]. Therefore, SDMs using bioclimatic variables alone are unable to account for many of the factors affecting the dispersal of species at landscape and regional scales, especially where landscapes are highly fragmented through clearing and/or land use modification [17]. In particular, SDMs are not able to incorporate the effects of altered demographic processes, barriers to movement and habitat degradation [18]. Furthermore, climate change may affect species behaviour and/or resource availability, thereby uncoupling existing relationships between predictive variables and potential distributions [19, 20]. Consequently, a SDM in a fragmented landscape will be of a greater utility value if it can demonstrate how taxa respond to fragmentation, or other potential impacts, in addition to responses to bioclimatic variables [17–19]. Case studies of taxa in fragmented landscapes are thus informative in investigating how model outputs can be enhanced with the addition of population-level data.

**Fig 1:**
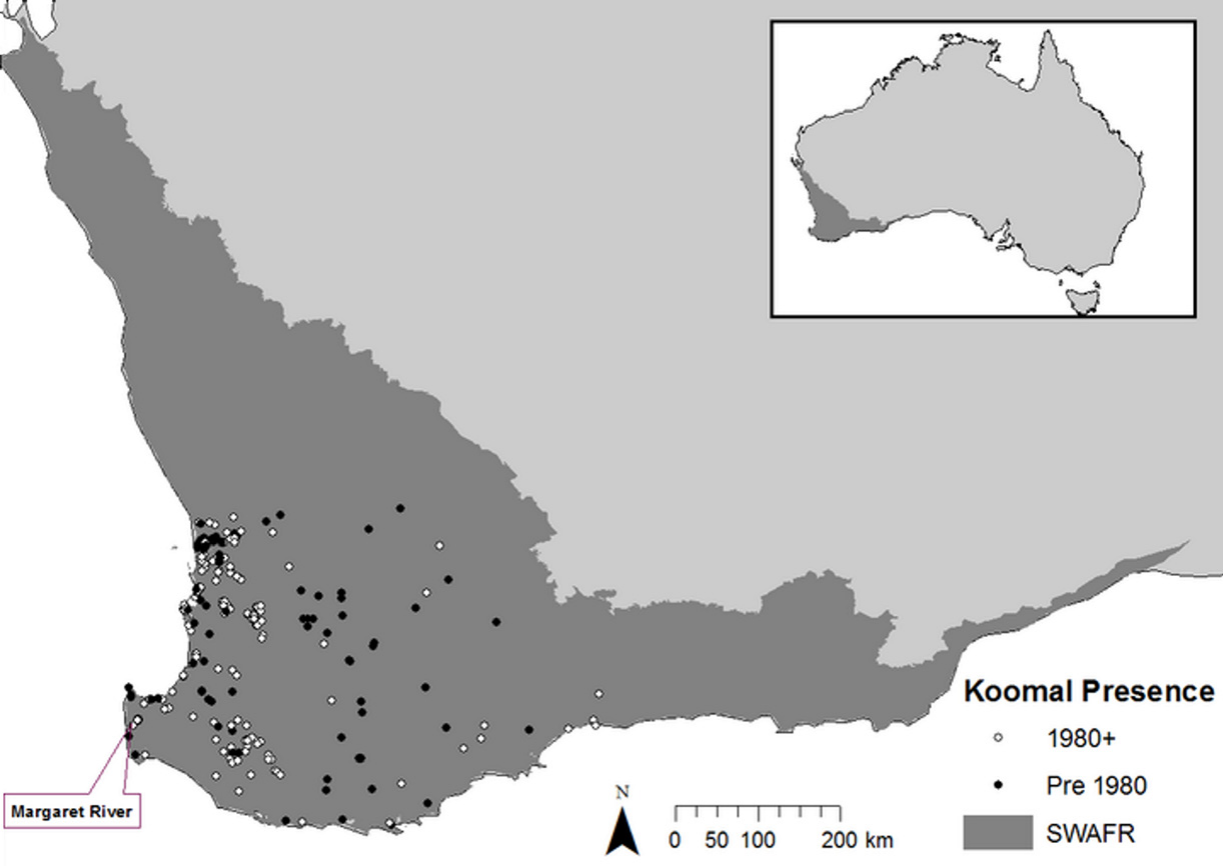
Modelled landscape with koomal presence. Pre and post 1980 presences recorded within the South-West Australian Floristic Region (SWAFR).

To demonstrate how field-based observations can be used to enhance a taxon-specific bioclimatic SDM, we use the example of the koomal (*Trichosurus vulpecula hypoleucus*). The koomal is a medium-sized arboreal marsupial endemic to south-western Australia. It is a geographically isolated and distinct sub-species of the common brushtail possum *T. vulpecula* that differs from other subspecies in terms of its smaller size, more omnivorous diet and denser fur [21, 22]. Since European settlement, the koomal has undergone a significant decline in both distribution and population size, now occupying less than 50% of its original distribution [23] (Fig 1). The reason for this reduction has largely been attributed to habitat loss, landscape fragmentation, habitat alteration/degradation and predation from the European red fox (*Vulpes vulpes*) and feral cat (*Felis catus*) [23, 24]. Another cause of loss of koomal habitat is jarrah dieback, caused by *Phytophthora* spp., soil-borne plant pathogens that kill or damage many overstorey and understorey plant species in the region [25].

A high level of land clearing has long been identified in the South West Australian Floristic Region (SWAFR). The Government of Western Australia [26] reported that there was less than 22% of native vegetation remaining on the southern Swan Coastal Plain and that some local government areas in the Western Australian Wheatbelt have less than 5% of native vegetation remaining. This contributes to a landscape where much of the native vegetation remaining exists within small, highly fragmented patches across most of the region, particularly in urban and agricultural areas.

The objectives of this paper are:

1. Use a case study of the koomal to demonstrate a means by which taxon-specific observations, gathered through an extensive trapping and radio tracking exercise, can add resolution and robustness to a SDM;
2. Demonstrate how the inclusion of taxon-specific variables can change SDM outputs and enhance the modelling process.

To do this, taxon-specific ecological variables will be used in conjunction with generic bioclimatic data to produce a potential distribution model for the koomal, which is then used to predict impacts of climate change using various emission scenarios. We then compare these predictions to those which based on models using just bioclimatic data.

## Materials and Methods

### Modelling Approach and Data

This paper relies heavily on the data published in Molloy [11]. These data are the product of a study which took place on three adjoining private properties in the Cowaramup catchment, situated in the Margaret River Region of Western Australia, with the full consent of all property owners involved. It was a twelve month field study undertaken to investigate how koomal responded to landscape fragmentation. Fieldwork involved capture, mark and release, and radio tracking activities run concurrently. Trapping was conducted on a seasonal basis with an average of three trapping periods per season with trapping being conducted over three to five consecutive nights for each period for a total of 52 nights. Traps were of the medium Sheffield wire cage type. Sixty one individual koomal were captured and micro-chipped (pouched young were not micro-chipped or counted) with a total of 360 captures. Of the animals captured, 31 were males and 30 females. Eleven koomal had Sirtrack VHF radio-tracking collars fitted at various stages throughout the project, ranging in periods from several weeks to the full length of the project. Collared animals were tracked one day per week to a daylight nesting site. All koomal were processed and released at point of capture and micro-chipped using Global-ident FDX-B transponders. All non-target species were immediately released without processing. All handling was undertaken by, or under the direct supervision of, an appropriately qualified and experienced wildlife handler in compliance with Edith Cowan University animal ethics approval 5669 and Department of Environment and Conservation licences SF007736 and SF008379.

All supporting data are available online within the above thesis at: http://ro.ecu.edu.au/cgi/viewcontent.cgi?article=1871&context=theses. Underlying data will be made available to all interested researchers upon request to the corresponding author. Geographic coordinates are not given for capture sites as all work was done on private property and some of the property owners wish to keep their anonymity.

Our approach was to use MaxEnt [27] to combine koomal presence data with remnant vegetation and bioclimatic data to ascertain the current potential distribution for the koomal and the predicted 2050 impacts of climate change on the potential distribution resulting from the application of selected GCMs. Geographical Information System (GIS) overlays were then used to explore how fragmentation and dieback might impact on those distributions.

We used MaxEnt as the principal SDM tool. Some drawbacks have been noted with MaxEnt, notably the tendency for it to underperform where there is a spatial bias within data sets [28]; however it remains a well-supported and popular application with land managers, and has the capacity to link fine-scale bioclimatic data to species distributions and produce probability-based outputs [1, 29, 30].

The koomal is arboreal and subject to an increased threat from introduced predators when on the ground. Consequently, we assumed that a SDM that uses bioclimatic variables alone and overlooked the impacts of fragmentation on this taxon would depict an unrealistic potential distribution as areas without sufficient vegetation would be depicted as habitat. To reflect how fragmentation affects the koomal, three GIS raster data sets were constructed from the Department of Agriculture and Food Western Australia’s 2013 remnant vegetation data set, namely:

1. 1 km^2^, i.e. the percentage of remnant vegetation cover within each pixel, with a pixel size of 1 km^2^ in size (this, coincidentally, is the approximate average diameter of koomal home ranges at their widest point as determined in the tracking analyses undertaken by Molloy [11].
2. 9 km^2^, i.e. the percentage of remnant vegetation cover within each pixel and within a 1 km radius of that pixel (including those adjoining diagonally), with a pixel size of 1 km^2^ (total area 9 km^2^). This scale is potentially relevant to home range movements as it is unlikely that a single 1 km^2^ pixel would exactly overlap similarly sized home range of an individual koomal. However, by buffering that pixel by a further 1km (the 9 km^2^ variable layer), we believe that there was a greater likelihood that all movements for a koomal identified in any individual pixel would be captured.
3. 25 km^2^, i.e. i.e. the percentage of remnant vegetation cover within a pixel size of 5 km x 5 km (total area 25 km^2^). This scale is potentially relevant to metapopulation movements.

Molloy [11] found a mean population size of 27.75 individuals present at any given trapping event across the 100 ha study area containing 41% remnant vegetation cover, with a total estimated population of 69 individuals having resided in this landscape during the study period. It was found that koomal distribution was patchy with, inexplicably, no animals caught in some habitat remnants. Gaps between remnants of up to 100 m presented no discernible barrier to movement and gaps of approximately 400 m were crossed regularly. Weights, sexual dimorphism and reproductive data, remained comparable with populations in contiguous, conservation-managed landscapes [11]. Kernel density estimates (at 95% probability) gave mean home ranges for males of 8.8 ha of remnant vegetation and 7.9 ha for females which varied from 0.3 to 1.1 km in width. Although subjects probably passed through areas heavily impacted by dieback, none were observed or captured in infested areas, indicating that dieback-affected areas were not suitable koomal habitat. As this is a binary response to a variable, unlike the probabilistic response to the availability of remnant vegetation (where the greater the amount of remnant vegetation in the landscape, the greater the probability of koomal presence), we decided to simply cut dieback infested areas from the predicted outputs of the MaxEnt SDM as the most accurate and simplest way of depicting the subject’s response to a binary variable.

### Model Construction

By using both baseline (1985–2005 averages) and climate change scenario data, we used MaxEnt to produce both a baseline SDM and an SDM that reflects the changes predicted in a future climate scenario. Baseline climate data were sourced from the WorldClim Coupled Model Intercomparison Project Phase 5 (CMIP5) database [31], developed as interpolated climate surfaces for global land areas other than Antarctica at a 1 km grid spatial resolution using 1950–2000 climate data as a baseline. These data were provided in the form of 19 bioclimatic variables (Table 1) [32] and clipped to the spatial extent shown in (Fig 1). This landscape was considered appropriate for modelling koomal potential distribution because all recorded presences fell within its boundaries and it is large enough to demonstrate a potential increase in distribution. The GCM used was the Australian Community Climate and Earth-System Simulator (ACCESS) 1.0 coupled model [33]. This model was chosen as, being a coupled product of the most reliable of the CMIP5 models in predicting the impacts of global warming in south-western Australia [34], it would provide the most likely climate change scenario. The 4.5 (medium) and 8.5 (high) representative concentration pathways (the greenhouse gas emission scenarios used in the CMIP 5 climate models) were chosen for this demonstration as recent studies show that these are considered the most probable outcomes [35]. Projections were made to the year 2070, as it assumed that more robust climate modelling would be available after this year and earlier scenarios may not provide an adequate indication of climate change impact on the koomal.

**Table 1:** Contribution of bioclimatic variables to the koomal SDM using all variables and five “minimum set” variables alone. * denotes variable used in minimum set.

Variable	Description	% Contribtion (all vars.)	% Contribtion (min. set)
Bio 1	Annual mean temperature	1.0	-
Bio 2	Mean diurnal range	2.5	-
Bio 3	Isothermality, i.e. (Bio1/Bio7)x100	0.6	-
Bio 4*	Temp. seasonality (coefficient of variation)	3.9	6.9
Bio 5	Max. temp. of warmest period	0.7	-
Bio 6	Min. temp. of warmest period	0.3	-
Bio 7	Temp. annual range	1.3	-
Bio 8	Mean temp. of wettest quarter	0.6	-
Bio 9	Mean temp. of driest quarter	0.9	-
Bio 10	Mean temp. of warmest quarter	3.7	-
Bio 11	Meant temp, of coldest quarter	0.3	-
Bio 12*	Annual precipitation	36.8	46
Bio 13	Precipitation of wettest period	2.4	-
Bio 14*	Precipitation of driest period	3.2	4.9
Bio 15*	Precipitation seasonality (coefficient of variation)	2.7	5.4
Bio 16	Precipitation of wettest quarter	3.9	-
Bio 17	Precipitation of driest quarter	1.0	-
Bio 18	Precipitation of warmest quarter	0.2	-
Bio 19*	Precipitation of coldest quarter	34.8	36.8

Table notes.Variable contribution to model using all variables and using the five “minimum set” variables alone.

To produce the dependant variable data set, 1,114 records of koomal presences were obtained from the Department of Parks and Wildlife’s NatureMap database [36]. For this exercise all pre-1980 records were removed as extensive land clearing throughout much of the koomal distribution during this period [37, 38] means that many of the woodlands from which presences were recorded have now been cleared. In other words, remnant vegetation cover for these recorded presences cannot be ascertained. This is also a risk for post-1980 records, but as land clearing has significantly slowed after 1980 [39], and as nearly all koomal records for this period can be attributed to existing native vegetation extent, we considered this impact to be, comparatively, marginal and offset by the rigour provided by a robust sample size of 918 presences.

Koomal presence records were shown to be heavily biased by ongoing trapping programs. For example, of the 918 presences, approximately 300 originated from the research undertaken by Molloy [11]. To mitigate this bias in testing the effectiveness of the three spatial variables, a low-bias sample was created [1, 40]. To do this, presences were consolidated into a one second (approximately1 km^2^) raster grid, the same scale as the bioclimatic variable data sets. Consequently presences are represented as either present or absent in each 1 km2 grid square, thereby providing a representation of koomal distribution based on 170 grid squares with greatly diminished trapping bias [41]. These raster data were then overlaid on the data sets representing the three spatial variables in a GIS environment and the results analysed using histograms, summary statistics and exploratory MaxEnt modelling to determine which data set was best suited for incorporation into the final MaxEnt model. The full presence only data set was retained for use in all MaxEnt models as the MaxEnt software automatically sorts presence data into presence by grid squares as part of the modelling process.

As there is a strong possibility that the use of any of the remnant vegetation data sets along with the full suite of bioclimatic variables may lead to “over-fitting” [29], it was decided to use the minimum number of bioclimatic variables required to give an Area Under Curve (AUC) training value greater than 0.955 before the inclusion of the remnant vegetation data. This figure was selected as any value above this indicates a very high level of model accuracy in comparison to the 0.5 null model result. Bioclimatic variables were removed from the SDM by conducting multiple model runs and removing the worst performing variables, according to “jacknife” analysis and % contribution to the SDM in each run. The model produced using the final suite of selected variables was compared with the original (19 variable) model to ensure that model integrity had not been compromised. When running the final model, a 20% training presence threshold was set and the process replicated ten times, with all other MaxEnt settings left as default values.

To incorporate the potential impacts of dieback on koomal potential distribution in the final model, a dieback distribution data set at an appropriate extent was required. Sub-regional dieback extent GIS shapefiles for the Northern, Southern, South-West and Swan NRM regions [42]] were sourced from Project Dieback [42] and adapted for inclusion in a MaxEnt model by merging these data sets, simplifying outputs to “probably present, probably absent and unknown,” and converting the resulting shapefile to an ASCII format. As Molloy [11]] found that the koomal response to dieback was binary, i.e. koomal were not observed in infected areas and therefore these areas are assumed to not be habitat. This data set was overlaid on the MaxEnt SDM thereby removing all infected areas from the koomal’s potential distribution.

Please note that model readouts, maps, and inputs are not necessary to replicate the findings and results given in this manuscript. Using the methods and (publicly available) data sets described in this manuscript will enable the reader to replicate the following results.

## Results

**Fig 2:**
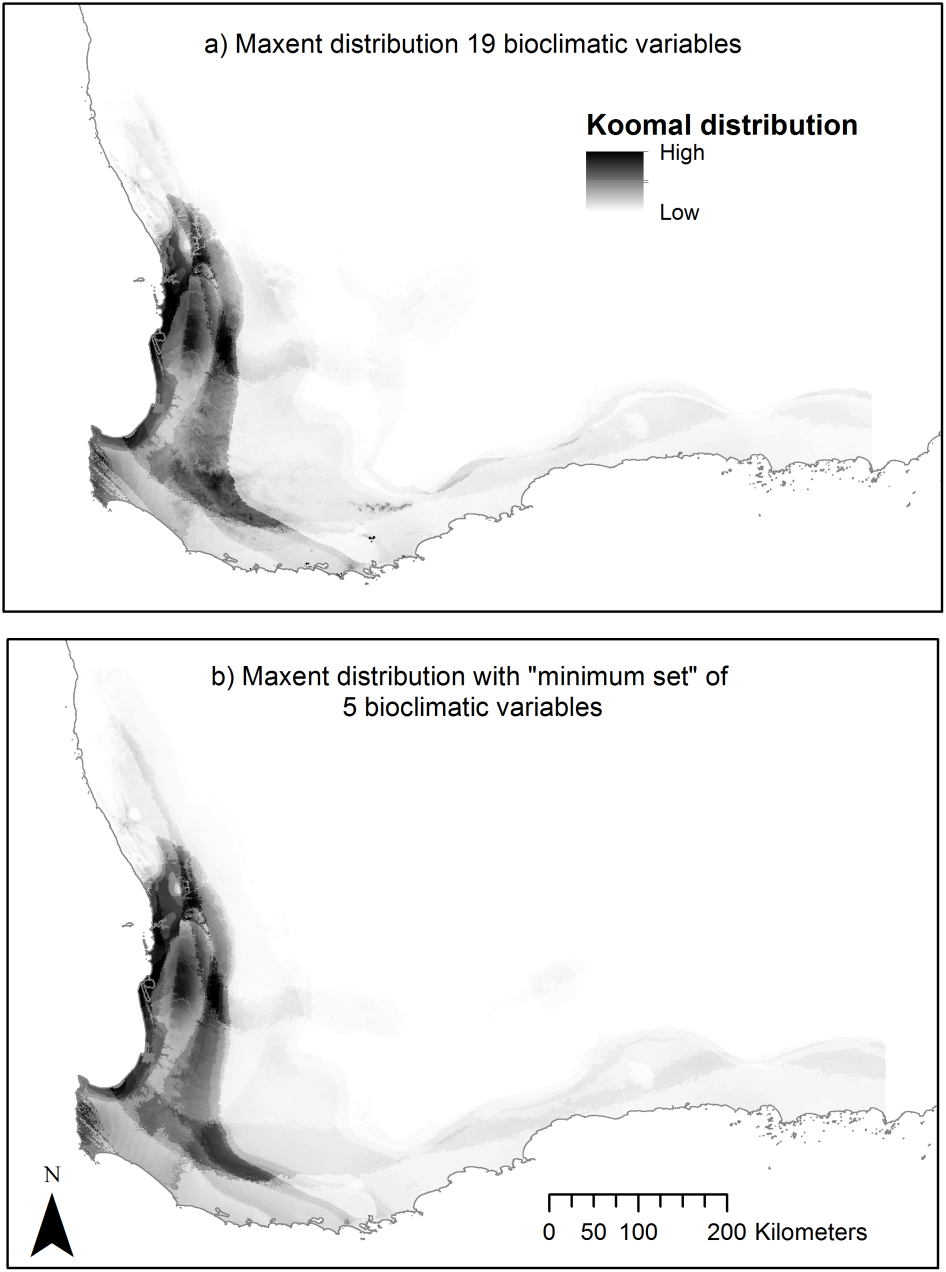
Comparison between MaxEnt koomal SDMs using full suite of 19 bioclimatic variables (a) and selected suite of 5 most significant variables (b). Lists of both sets of variables are given in Table 1.

By running the post-1980 koomal presence records using all 19 bioclimatic variables, removing variables with a contribution less than 1%, and repeating the process, a minimum set of five bioclimatic variables were selected for use in this exercise (Table 1). Although this resulted in a very small reduction in the average training AUC value from 0.972 to 0.965, the change in the average test AUC value from 0.968 to 0.947 and the increase in 10% threshold area from 7.84% of the landscape to 9.65%, indicated both SDMs are very robust and the geographic differences in the potential distributions were negligible (Fig 2). Furthermore, the potential problems associated with over-fitting in further modelling scenarios were greatly reduced.

**Fig 3:**
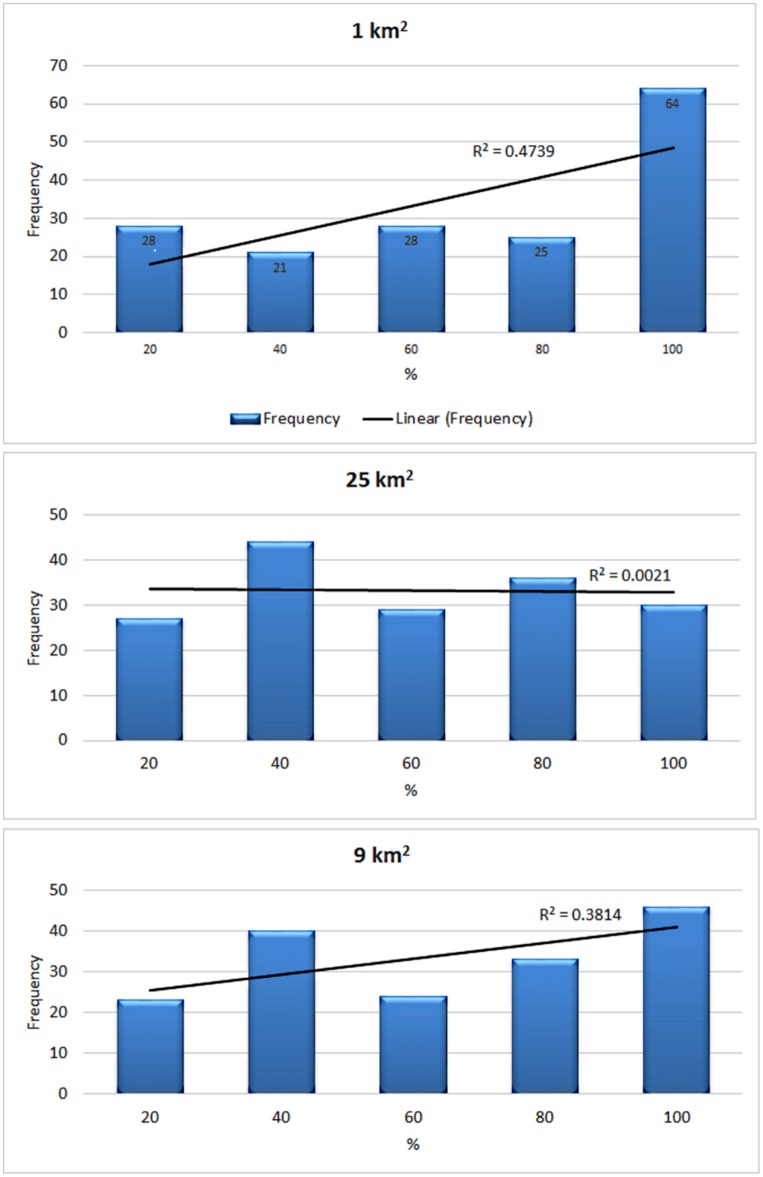
Histograms and line representing the response of koomal to the proportion of remnant vegetation cover as calculated from the three spatial variables. R^2^ shows fit to linear model using raw data.

To determine which of the spatial variables was the most effective predictor, the low bias sample of post 1980 koomal presences were overlaid over each data set. Resulting histograms indicated that koomal had a preference for a) full vegetation cover at the km^2^ variable; b) a relatively flat response with a slight preference for approximately 40% of remnant vegetation cover for the 25 km^2^ variable; and c) a less pronounced preference for full vegetation cover with another spike at 40% for the 9 km^2^ variable (Fig 3).

Although summary statistics (Table 2) showed no appreciable difference between the three spatial variables in terms of mean, standard deviation, and median, with the 1 km^2^ scoring the highest, the 25 km^2^ the lowest and the 9 km^2^ in between them. However, the 9 km^2^ perspective was much less skewed (Fig 3, Table 2).

**Table 2:** Summary statistics for the response of koomal to the proportion of remnant vegetation cover as calculated from the three variables given in Fig 3.

Test	1 km^2^	25 km^2^	9 km^2^
Mean	60.19	50.65	55.53
Standard error	2.58	2.14	2.27
Median	65.69	45.98	56.95
Standard dev.	33.08	27.48	29.3
Sample variance	1094.14	754.91	858.29
Kurtosis	-1.26	-1.07	-1.3
Skewness	0.34	0.288	-0.06
Range	100	94.38	99.9
Min.	0	4.65	0.1
Max.	100	99.02	100
Sum	9932.02	8365.99	9218.05
Count	165	165	166
Confidence level (95%)	5.08	4.22	4.49

Table note. As calculated from the three perspectives given in Fig 3.

When incorporated into the MaxEnt SDMs, there was little difference in statistical tests of model accuracy between the model with no added spatial variables and those with the remnant vegetation variable data sets added (Table 3). In this exercise it was shown that Area Under Curve (AUC) values for the models run with the 25 km^2^ and the 9 km^2^ data sets were marginally superior to the model run without any spatial variables, with the rankings, contribution and 10% threshold values of these two models being superior to the model run with the 1 km^2^ variable. In all values, the 25 km^2^ and 9 km^2^ variables remained very similar with the 25 km^2^ variable scoring slightly higher in all indicators.

**Table 3:** Model accuracy indicators with remnant vegetation perspective data sets added.

Landscape variable	AUC	Ranking (1–6)	Contribution (%)	10% Threshold
None	0.965	-	-	0.229
1 km^2^	0.965	5	5.3	0.182
9 km^2^	0.967	3	8.5	0.221
25 km^2^	0.967	3	10.3	0.226

Table note. AUC is a statistical test undertaken by the MaxEnt software. (A perfect model will score an AUC of 1, while random guessing will score an AUC of around 0.5). Ranking is the comparative importance of the variable when added to the SDM. Contribution is the contribution of the variable when incorporated into the SDM. The 10% threshold is a cut off value used to convert the output from probabilistic to binary. It is determined as the probability value above which 90% of presences can be found.

**Fig 4:**
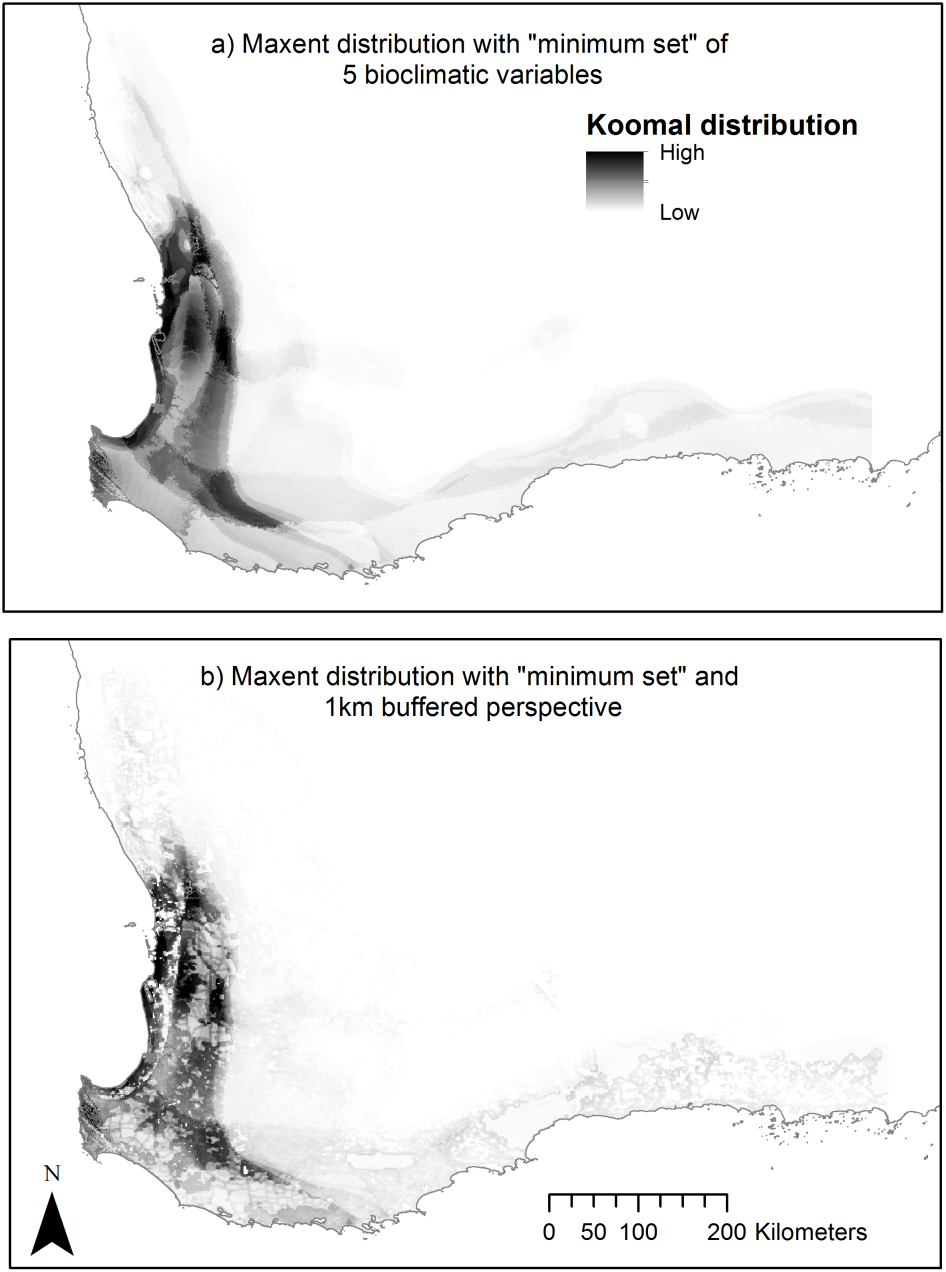
MaxEnt PD calcualted with (a) the minimum set of 5 bioclimatic variables alone and (b) with the minimum set and 9 km^2^ variable model (the baseline model).

The comparison of the MaxEnt model with the reduced set of five bioclimatic variables with that of the model incorporating the 9 km2 remnant vegetation variable (Fig 4), found that both potential distributions remained generally similar in probability of presence and extent. The most notable difference between these SDMs was the more appropriate level of detail evident where the 9 km^2^ variable was included in the model. This reflects the percentage of remnant vegetation within 1 km radius of each pixel, and by extension, the level of landscape fragmentation. It also demonstrates the koomal’s response to that variable. By removing less suitable habitats from the model in this way, the SDM has been made more capable of quantifying habitat at a finer scale than the SDM constructed with bioclimatic variables alone. This is further confirmed by comparing the average 10% thresholded areas for both SDMs, where the threshold area for the minimum set model is 9.65% of the modelled area compared to the area with the 9 km^2^ variable of 8.22%. This significant reduction in area (1.4% of total area or an 11.7% reduction in selected area) is largely achieved by omitting areas that have low habitat values. For these reasons, this model (Fig 4b) was used as the baseline for modelling climate change impacts.

**Fig 5:**
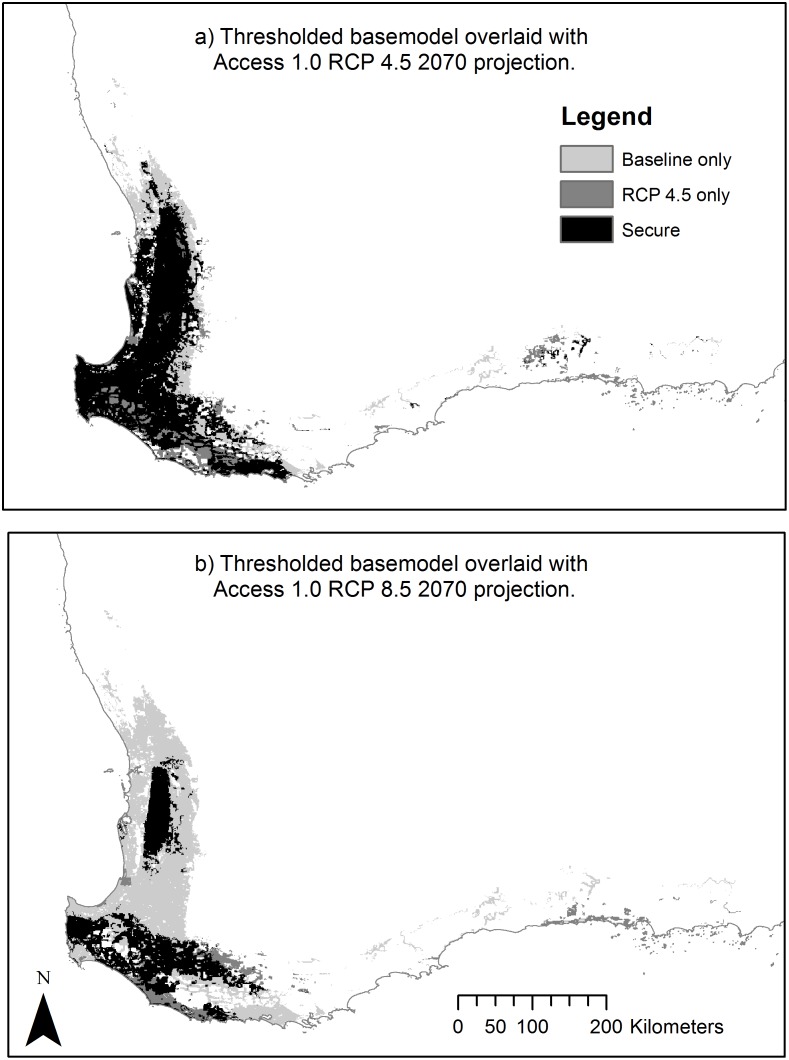
Overlays showing baseline with RCP 4.5 (a) and RCP 8.5 (b) projected distributions overlaid to show areas identified in baseline and projected distributions and secure areas. Secure areas being those that are recognised as current potential distribution and which are predicted to remain as such.

A comparison of predictions from the baseline model (Fig 4b) with models incorporating climate change impacts as predicted in the ACCESS 1.0, RCP 4.5 and 8.5 2070 GCMs. To do this, models outputs were changed from probabilistic to binary by arbitrarily applying cut-off thresholds at a fixed cumulative value of 10% (Fig 5).

**Fig 6:**
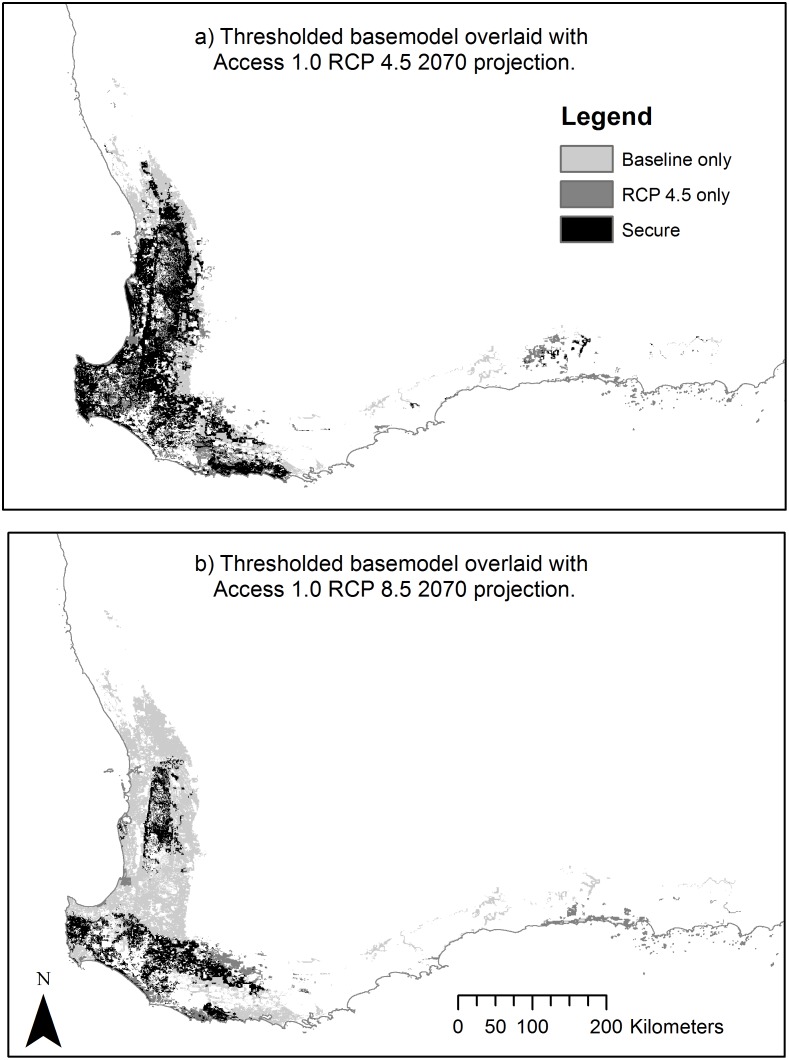
a) RCP4.5 and b) RCP 8.5 2070 SDMs with jarrah dieback infested areas removed.

When areas where dieback is, or is expected to be, present were overlaid over both RCP 4.5 and 8.5 projections (Fig 6) clear reductions in likely potential distributions were demonstrated. This exercise shows that dieback substantially reduces potential distributions in baseline and in both 2070 potential distributions, even at the current level of dieback infestation.

## Discussion

**Fig 7:**
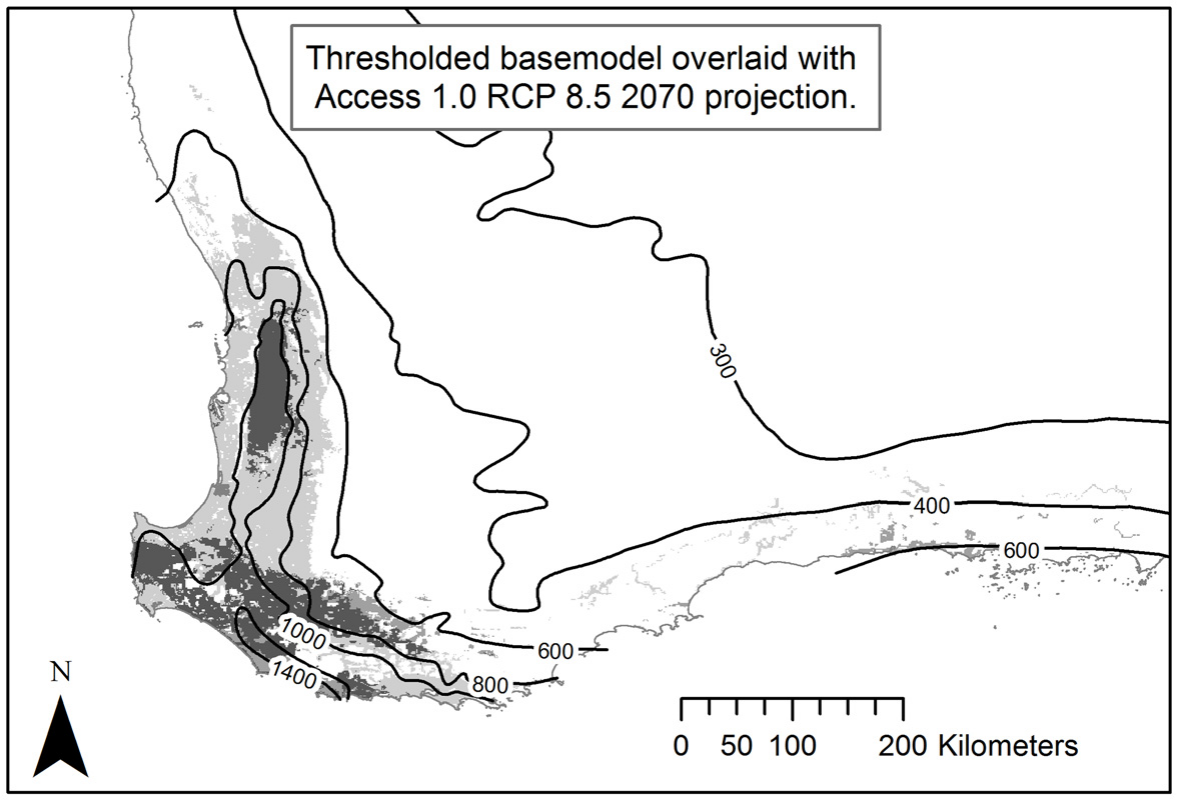
RCP 8.5 2070 projection of koomal distribution (Fig 6b) with current annual average precipitation (mm) overlaid.

The MaxEnt SDM constructed with bioclimatic variables alone demonstrates a contraction in potential distribution similar to those predicted by SDMs for other medium-sized marsupials in this region, i.e. the ngwayr [43] and quokka [10]. This is to be expected as all three taxa share a common distribution and because all of these models are strongly influenced by precipitation variables. This is not surprising given the Mediterranean-type climate with highly seasonal winter-dominated rainfall [12, 44] that has been shown to be consistently and significantly declining over recent decades [45]. The 2070 RCP 8.5 model shows that koomal potential distribution will be split into two distinct, northern and southern, populations (Fig 6b). Such a split in habitat was also predicted for the marri (*Corymbia calophylla*) and jarrah (*Eucalyptus marginata*) [43], these are two dominant tree species upon which the koomal is largely reliant on for habitat [21]. This reflects the predicted core distribution of all of these taxa within a relatively small area where precipitation is currently greater than 650 mm. The ACCESS 1.0 RCP 8.5 2070 projection predicts the area within this rainfall isohyet will contract by approximately 65% to the area that approximates the current >900 mm rainfall isohyet. This is similar to the contraction predicted by the koomal SDM for this climate projection (Fig 7).

A modelling study on another possum species in the region, the ngwayir [43], used a suite of 19 bioclimatic predictive variables even though this represented a light risk of over-fitting. [29, 46, 47]. This was done because, in that exercise, the capacity of MaxEnt itself was being tested and because this model was being used to test the response of other species that define its habitat, all of which may have had different bioclimatic requirements. In this current study, where only one taxon was being modelled and additional habitat variables were used, it was decided to select a suite of five bioclimatic variables to remove potential bias and overfitting, and to add resolution to the modelling process [48, 49]. This was done by undertaking multiple model runs and removing the worst performing variables. This produced a koomal post-1980 potential distribution model with very little visual or statistical difference from the model output where all 19 bioclimatic variables were used.

By applying remnant vegetation mapping at the most appropriate scale to produce a predictive variable data set and incorporating this into the SDM, it was possible to take broad bioclimatic potential distributions and apply those onto actual landscape attributes. To do this, two issues had to be resolved. The first required finding a quantifiable landscape parameter that reflected a koomal habitat preference, in this case remnant vegetation cover. The second was finding an appropriate spatial variable from which to view that habitat parameter. To do this, field observations were used to hypothesise a group of probable variables that could then be tested to determine which of these was the most suitable.

The 9 km^2^ spatial variable proved to be the most useful within the context of defining habitat for this taxon in this landscape. Although, statistically, it appeared in some criteria to be marginally less effective than the 25 km^2^ variable, it better addressed two important criteria for model success: 1) it quantified an important habitat parameter; and, 2) through providing better resolution, the model’s utility value was enhanced [50].

The results of this exercise did not statistically detract from the bioclimatic modelling, as AUC values remain relatively constant regardless of which landscape variable is added (Table 3). Nor do they change the broad extent of koomal’s potential distribution. However, the inclusion of a GIS laye that reflects the koomal’s habitat preferences into the modelling process lowers the probability of presence for areas within that extent that, by virtue of their lack of remnant vegetation, are quantifiably less likely to support koomal. Furthermore, the probability value of areas with adequate vegetation cover remain unchanged. Consequently, it can be safely assumed that this the inclusion of such a habitat specific variable into the model will enhance the modelling process by improving its capacity to identify and prioritise core habitat areas.

Molloy [11]found that areas infested with dieback were not koomal habitat. By overlaying the dieback extent data set it was possible to eliminate areas that were, or probably were, not habitat from model outputs. This also helps to refine the delivery of targeted conservation management activities. Of note in this exercise was the finding that dieback reduces the post-1980 core potential distribution of koomal by 18% and the 2050 core potential distribution by 28% (compared to current extent). It also highlighted that the area of 2050 potential distribution will be reduced by 36% and the areas identified as core potential distribution by all three GCMs declined by 51%. This example shows how those areas most likely to provide core habitat for the koomal in the future are the same areas at greatest risk from dieback. This suggests that these areas should not only be maintained as koomal habitat, but also that rehabilitation of dieback-impacted areas, as well as further *Phytophthora spp*. mapping and quarantining of this pathogen where it is absent [51], should be undertaken in these areas as a matter of priority.

There are no available data predicting remnant vegetation extent or the potential extent of dieback *circa* 2050 for the modelled landscape. For this reason, post-1980 and -2050 models were run using the current data set. Although this allowed the benefits of including taxon-specific data in potential distribution SDMs to be demonstrated, it does present a potential problem in applying the findings of this modelling exercise to conservation management. Therefore, it is recommended that the outputs of this exercise be viewed with this limitation acknowledged, especially as the impacts of climate change on dieback are also poorly understood. Given that the distribution, rate of dispersal and impact of this fungal pathogen are related to bioclimatic variables in general and precipitation in particular [40, 42, 51], it is likely that climate change may bring about a change in dispersal, or even a range contraction, for this pathogen.

Our work here subscribes to the call to improve SDMs predicting the impact of climate change by incorporation of information on the ecology of the species and finer-scale habitat variables with traditional bioclimatic ones [2, 3, 52]. Similar improvements were made by Fordham et al [53] who added geological substrate, topography and distribution of grassland habitat (via two dominant plant species) to bioclimatic modelling of the pygmy blue-tongue lizard*Tiliqua adelaidensis* in South Australia, and by Adams-Hosking et al [15] who improved predictions of koala *Phascolarctos cinereus* decline by adding the predicted distributions of the *Eucalyptus spp*. that they feed on to their SDMs. We acknowledge that the species-specific variables used in this SDM have been selected to reflect field observations of a specific taxon within the context of a specific landscape. Therefore, our specific variables and SDMs may not be able to be directly applied to other taxa, particularly those facing different threatening processes. In this study we have demonstrated the principle of incorporating the findings of field research into the modelling process and, in so doing, adding a valuable level of rigour to the modelling process thereby imporving their predictive ability and resolution.

## Conclusions

This study demonstrates a means by which taxon-specific observations gathered through intensive fieldwork can be used to add resolution and robustness to a SDM. We have demonstrated how spatial modelling can be used to model a future potential distribution for a taxon that has been shown to be vulnerable to the impacts of landscape fragmentation, a rapidly changing climate and *Phytophthora spp*. a virulent plant pathogen that impacts negatively on koomal habitat.

In comparison to the simple bioclimatic model developed for another possum in the region [43], the incorporation of landscape data enabled the development of a much more realistic and finer resolution potential distribution. By combining the habitat preferences of the koomal with bioclimatic parameters, areas that are not likely to be considered habitat by virtue of an unsuitable landscape matrix, or because of *Phytophthora spp*. infestation, were removed from the potential distribution whilst largely retaining the habitat values of the bioclimatic-only model. A comparison between these two outputs will help to inform those areas where management activities such as dieback control, a change in tenure or revegetation could best be undertaken to help assure the persistence of this taxon. It also highlights those areas that, although not currently koomal habitat, may become so in the future.

## References

[1] Hijmans RJ, Graham CH. The ability of climate envelope models to predict the effect of climate change on species distributions. Global Change Biology. 2006;12(12):2272–81. 10.1111/j.1365-2486.2006.01256.x

[2] Guisan A, Thuiller W. Predicting species distribution: offering more than simple habitat models. Ecology Letters. 2005;8(9):993–1009. 10.1111/j.1461-0248.2005.00792.x 34517687

[3] Franklin J. Mapping Species Distributions: Spatial Inference and Prediction. Cambridge: Cambridge University Press; 2010.

[4] Schröter M, Remme RP, Sumarga E, Barton DN, Hein L. Lessons learned for spatial modelling of ecosystem services in support of ecosystem accounting. Ecosystem Services. 2014.

[5] Meineri E, Deville AS, Grémillet D, Gauthier-Clerc M, Béchet A. Combining correlative and mechanistic habitat suitability models to improve ecological compensation. Biological Reviews. 2015;9:314–29. 10.1111/brv.12111 24837691

[6] Brotons L. Species Distribution Models and Impact Factor Growth in Environmental Journals: Methodological Fashion or the Attraction of Global Change Science. PloS one. 2014;9(11):e111996 10.1371/journal.pone.0111996 25386926 PMC4227683

[7] Araújo MB, Guisan A. Five (or so) challenges for species distribution modelling. Journal of Biogeography. 2006;33(10):1677–88. 10.1111/j.1365-2699.2006.01584.x

[8] Booth TH, Nix HA, Busby JR, Hutchinson MF. BIOCLIM: the first species distribution modelling package, its early applications and relevance to most current MAXENT studies. Diversity and Distributions. 2014;20(1):1–9. 10.1111/ddi.12144

[9] Radosavljevic A, Anderson RP. Making better Maxent models of species distributions: complexity, overfitting and evaluation. Journal of biogeography. 2014;41(4):629–43. 10.1111/jbi.12227

[10] Gibson L, McNeill A, de Tores P, Wayne A, Yates C. Will future climate change threaten a range restricted endemic species, the quokka (Setonix brachyurus), in south west Australia? Biological Conservation. 2010;143(11):2453–61. PubMed PMID: ISI:000283412300005. 10.1016/j.biocon.2010.06.011

[11] Molloy S. Applying the principles of spatial modelling to the management of biodiversity in the fragmented landscapes of south-western Australia [Thesis]: Edith Cowan University; 2013.

[12] Yates CJ, Elith J, Latimer AM, Le Maitre D, Midgley GF, Schurr FM, et al Projecting climate change impacts on species distributions in megadiverse South African Cape and Southwest Australian Floristic Regions: Opportunities and challenges. Austral Ecology. 2010;35(4):374- 10.1111/j.1442-9993.2009.02044.x

[13] Prober SM, Lemson K, Lyons T, Macfarlane C, O’Connor MH, Scott JK, et al Facilitating adaptation of biodiversity to climate change: a conceptual framework applied to the world’s largest Mediterranean-climate woodland. Climatic Change. 2012;110(1):227–48. 10.1007/s10584-011-0092-y

[14] Elith J, Kearney M, Phillips S. The art of modelling range-shifting species. Methods in Ecology and Evolution. 2010;1(4):330–42. 10.1111/j.2041-210X.2010.00036.x

[15] Adams-Hosking C, Grantham HS, Rhodes JR, McAlpine C, Moss PT. Modelling climate-change-induced shifts in the distribution of the koala. Wildlife Research. 2011;38(2):122–30. 10.1071/WR10156

[16] Elith J, Leathwick JR. Species distribution models: ecological explanation and prediction across space and time. Annual Review of Ecology, Evolution, and Systematics. 2009;40(1):677 10.1146/annurev.ecolsys.110308.120159

[17] Urban MC, Zarnetske PL, Skelly DK. Moving forward: dispersal and species interactions determine biotic responses to climate change. Annals of the New York Academy of Sciences. 2013;1297(1):44–60. 23819864 10.1111/nyas.12184

[18] Hand BK, Cushman SA, Landguth EL, Lucotch J. Assessing multi-taxa sensitivity to the human footprint, habitat fragmentation and loss by exploring alternative scenarios of dispersal ability and population size: a simulation approach. Biodiversity and Conservation. 2014;23(11):2761–79. 10.1007/s10531-014-0747-x

[19] Huntley B, Barnard P, Altwegg R, Chambers L, Coetzee BWT, Gibson L, et al Beyond bioclimatic envelopes: dynamic species’ range and abundance modelling in the context of climatic change. Ecography. 2010;33(3):621–6.

[20] Willis SG, Thomas CD, Hill JK, Collingham YC, Telfer MG, Fox R, et al Dynamic distribution modelling: predicting the present from the past. Ecography. 2009;32(1):5–12. 10.1111/j.1600-0587.2008.05711.x

[21] Cruz J, Sutherland DR, Martin GR, Leung LKP. Are smaller subspecies of common brushtail possums more omnivorous than larger ones? Austral Ecology. 2012;37(8):893–902. 10.1111/j.1442-9993.2011.02346.x

[22] Kerle JA, Mckay GM, Sharman GB. A Systematic Analysis of the Brushtail Possum, Trichosurus-Vulpecula (Kerr, 1792) (Marsupialia, Phalangeridae). Australian Journal of Zoology. 1991;39(3):313–31. 10.1071/ZO9910313

[23] Jones B. The Possum Fauna of Western Australia: Decline Persistence and Status. In: Goldingay RL, Jackson SM, editors. The Biology of Australian Possums and Gliders. Chipping Norton: Surrey Beatty & Sons; 2004 p. 149–60.

[24] How RA, Hillcox SJ. Brushtail possum populations in south-western Australia: demography, diet and conservation status. Wildlife Research. 2000;27(1):81–9. 10.1071/WR98064

[25] Anderson P, Brundrett M, Grierson P, Robinson R. Impact of severe forest dieback caused by Phytophthora cinnamomi on macrofungal diversity in the northern jarrah forest of Western Australia. Forest Ecol Manag. 2010;259(5):1033–40. 10.1016/j.foreco.2009.12.015

[26] Government of Western Australia. 2013 Statewide Vegetation Statistics incorporating the CAR Reserve Analysis (Full Report). Current as of June 2013 Perth: WA Department of Parks and Wildlife; 2013 [12/05/2015]. Available from: https://www2.landgate.wa.gov.au/web/guest/downloader.

[27] Phillips SJ, Anderson RP, Schapire RE. Maximum entropy modeling of species geographic distributions. Ecological Modelling. 2006;190(3):231–59. 10.1016/j.ecolmodel.2005.03.026

[28] Bystriakova N, Peregrym M, Erkens RHJ, Bezsmertna O, Schneider H. Sampling bias in geographic and environmental space and its effect on the predictive power of species distribution models. Systematics and Biodiversity. 2012;10(3):305–15. 10.1080/14772000.2012.705357

[29] Elith J, Phillips SJ, Hastie T, Dudík M, Chee YE, Yates CJ. A statistical explanation of MaxEnt for ecologists. Diversity and Distributions. 2011;17(1):43–57. 10.1111/j.1472-4642.2010.00725.x

[30] Vasconcelos TS, Rodríguez MÁ, Hawkins BA. Species distribution modelling as a macroecological tool: a case study using New World amphibians. Ecography. 2012;35(6):539–48. 10.1111/j.1600-0587.2011.07050.x

[31] WorldClim. Global Climate Data Base 2014 [18 October 2014]. Available from: http://worldclim.org/.

[32] Hijmans RJ, Cameron SE, Parra JL, Jones PG, Jarvis A. Very high resolution interpolated climate surfaces for global land areas. International Journal of Climatology. 2005;25(15):1965–78. 10.1002/joc.1276

[33] Bi D, Dix M, Marsland SJ, O’Farrell S, Rashid H, Uotila P, et al The ACCESS coupled model: description, control climate and evaluation. Aust Meteorol Oceanogr J. 2013;63(1):41–64.

[34] Hope P. Southern and South-Western Flatlands Cluster Report. Climate Change in Australia Projections for Australia’s Natural Resource Management Regions: Cluster Reports, eds. Ekström, M. et al., CSIRO and Bureau of Meteorology, Australia; 2015.

[35] Kim HM, Webster PJ, Curry JA. Evaluation of short-term climate change prediction in multi-model CMIP5 decadal hindcasts. Geophysical Research Letters. 2012;39(10). 10.1029/2012GL051644

[36] DEC. NatureMap: Mapping Western Australia’s Biodiversity: Department of Environment and Conservation 2007- [13 October 2012]. Available from: http://naturemap.dec.wa.gov.au/.

[37] Hobbs RJ. Fragmented landscapes in Western Australia: Introduction. Biological Conservation. 1993;64(3):183–4. 10.1016/0006-3207(93)90319-V

[38] Saunders DA. Problems of survival in an extensively cultivated landscape: the case of Carnaby’s cockatoo Calyptorhynchus funereus latirostris. Biological Conservation. 1990;54(3):277–90. 10.1016/0006-3207(90)90057-V

[39] Office of the Auditor General. Management of native vegetation clearing / Auditor General for Western Australia2007.

[40] Phillips SJ, Dudík M. Modeling of Species Distributions with Maxent: New Extensions and a Comprehensive Evaluation. Ecography. 2008;31(2):161–75. 10.1111/j.0906-7590.2008.5203.x

[41] Giovanelli JGR, de Siqueira MF, Haddad CFB, Alexandrino J. Modeling a spatially restricted distribution in the Neotropics: How the size of calibration area affects the performance of five presence-only methods. Ecological Modelling. 2010;221(2):215–24. PubMed PMID: ISI:000273628800010. 10.1016/j.ecolmodel.2009.10.009

[42] Strelein G, Swain D, Miller J, Ball S, Green M. SCRIPT Dieback Risk Analysis. Pytophthora cinnamomi: Mapping the Threats and Building the Capacity to Manage Them. Perth: Department of Conservation and Land Management Western Australia, 2007.

[43] Molloy SW, Davis RA, Van Etten EJB. Species distribution modelling using bioclimatic variables to determine the impacts of a changing climate on the western ringtail possum (*Pseudocheirus occidentalis*; Pseudocheiridae). Environmental Conservation. 2014;41(2):176 10.1017/S0376892913000337

[44] Hearn R, Williams K, Comer S, Beecham B. JF2 -Southern Jarrah Forest subregion. In: McKenzie N, May J, editors. A biodiveristy audit of Western Australia’s biogeographical subregions in 2002. Kensington, W.A.: Department of Conservation and Land Mangagment; 2003 p. 382–403.

[45] Indian Ocean Climate Initiative. Western Australia’s weather and climate: a synthesis of Indian Ocean climate initiative stage 3 research. Australia: CSIRO and BoM, 2012.

[46] Benito BM, Martinez-Ortega MM, Munoz LM, Lorite J, Penas J. Assessing extinction-risk of endangered plants using species distribution models: a case study of habitat depletion caused by the spread of greenhouses. Biodiversity and Conservation. 2009;18(9):2509–20. PubMed PMID: ISI:000268190300016. 10.1007/s10531-009-9604-8

[47] Fitzpatrick MC, Gove AD, Sanders NJ, Dunn RR. Climate change, plant migration, and range collapse in a global biodiversity hotspot: the Banksia (Proteaceae) of Western Australia. Global Change Biology. 2008;14(6):1337 10.1111/j.1365-2486.2008.01559.x

[48] Hijmans RJ. Cross-validation of species distribution models: removing spatial sorting bias and calibration with a null model. Ecology. 2012;93(3):679–88. 10.1890/11-0826.1 22624221

[49] Warren DL, Seifert SN. Ecological niche modeling in Maxent: the importance of model complexity and the performance of model selection criteria. Ecological Applications. 2011;21(2):335–42. 10.1890/10-1171.1 21563566

[50] Šímová P, Gdulová K. Landscape indices behavior: A review of scale effects. Applied Geography. 2012;34(Journal Article):385–94.

[51] Shearer BL, Tippett JT. Jarrah dieback: the dynamics and management of Phytophthora cinnamomi in the jarrah (*Eucalyptus marginata*) forest of south-western Australia: Department of Conservation and Land Management Perth; 1989.

[52] Austin MP, Van Niel KP. Improving species distribution models for climate change studies: variable selection and scale. Journal of Biogeography. 2011;38(1):1–8. 10.1111/j.1365-2699.2010.02416.x

[53] Fordham DA, Watts MJ, Delean S, Brook BW, Heard L, Bull C. Managed relocation as an adaptation strategy for mitigating climate change threats to the persistence of an endangered lizard. Global change biology. 2012;18(9):2743–55. 10.1111/j.1365-2486.2012.02742.x 24501053

